# ARSENIC: In Search of the Roots of a Scourge

**DOI:** 10.1289/ehp.118-a20

**Published:** 2010-01

**Authors:** Carol Potera

**Affiliations:** **Carol Potera**, based in Montana, has written for *EHP* since 1996. She also writes for *Microbe*, *Genetic Engineering News*, and the *American Journal of Nursing*

In Bangladesh as many as 57 million people drink water with arsenic levels that exceed the 10-ppb limit set by the World Health Organization. Arsenic occurs naturally in high concentrations in groundwater in Bangladesh. Researchers at the Massachusetts Institute of Technology (MIT) in Cambridge and Bangladesh University of Engineering and Technology in Dhaka now report that ponds resulting from land excavation to build up villages may worsen arsenic contamination in local drinking water, whereas irrigated rice fields appear to remove arsenic.

Land use in Bangladesh has changed dramatically over the past few decades. Population growth has spurred land excavations to elevate homes and roadways for protection against monsoon floodwaters. The resulting pits fill with water and become fish ponds, which are rich in organic carbon that percolates downward. Iron-reducing bacteria such as *Geobacter* species transform solid-phase iron into soluble ferrous iron. Solid-phase iron binds arsenic; however, when bacteria transform the metal ions into ferrous iron, the bound arsenic is released into groundwater. Study leader Charles Harvey, an associate professor of civil and environmental engineering at MIT, believes these fish ponds may be a major contributor to the arsenic poisoning that is now endemic to Bangladesh.

People in Bangladesh once drank stagnant surface pond water and, as a result, were plagued by gastrointestinal infections. About 40 years ago, they switched to drilling shallow tube wells to obtain cleaner drinking water, with the number of wells doubling roughly every 5 years, according to Alexander van Geen, a geochemist at Columbia University who also has studied the Bangladeshi situation. Symptoms of arsenic poisoning, such as characteristic skin lesions, started showing up about 20 years ago.

In the current study, Harvey and colleagues monitored the hydrology and chemical composition of water from ponds and rice fields that recharge underground aquifers in a 9-km^2^ test site in the Munshiganj district of Bangladesh. Their measurements over 7 years included concentrations of arsenic, pH, oxygen, and organic carbon in water samples collected at different sites and water depths. They created a three-dimensional model to track water movement underground.

The team discovered that arsenic levels peaked about 30 m below ground level—the same depth to which many tube wells are drilled for drinking water. Moreover, when water was pumped out of aquifers to irrigate rice fields, the pond water was drawn down to about this same depth of 30 m, gaining arsenic as it passed downward through sediment layers. Atmospheric oxygen as well as that produced by algae in rice fields oxidized iron, causing it “to coagulate and settle out,” explains Harvey, absorbing arsenic as it did so. Chemical fingerprints of water samples collected at different locations confirmed that water with the highest arsenic content originated from human-built ponds while water coming from irrigated rice fields had the lowest arsenic content. The results were reported online 15 November 2009 ahead of print in *Nature Geoscience*.

The study was carefully conducted and is a valuable contribution, says van Geen. He is less certain that the organic matter transported into the aquifer through the bottom of ponds drives the reducing conditions that produce the arsenic peak in Bangladesh. “My colleagues and I believe organic matter buried in the sediment is a more likely source, although no one can really claim this has been demonstrated,” he says. “We believe the arsenic problem predates any significant human intervention—what has changed is that people didn’t drink a lot of groundwater in the area more before thirty years ago.”

Harvey next plans to dig 150-m wells for several families to share. If the water proves arsenic-free, he and colleagues will study whether drinking clean water flushes arsenic from the body or reduces skin lesions and other toxicity symptoms. “Most studies look at how arsenic causes health problems,” he says, “but this will be the reverse situation.”

